# Biomass-Templated Fabrication of Metallic Materials for Photocatalytic and Bactericidal Applications

**DOI:** 10.3390/ma12081271

**Published:** 2019-04-18

**Authors:** Xueying Guo, Qianqian Wang, Qiongyu Lai, Qiran Ouyang, Peng Li, Hai-Dong Yu, Wei Huang

**Affiliations:** 1 Institute of Advanced Materials (IAM) & Key Laboratory of Flexible Electronics (KLOFE), Jiangsu National Synergetic Innovation Center for Advanced Materials (SICAM), Nanjing Tech University (NanjingTech), Nanjing 211816, China; iamxyguo@njtech.edu.cn (X.G.); qqwang0717@njtech.edu.cn (Q.W.); laiqionyu03@njtech.edu.cn (Q.L.); iamoyqr@njtech.edu.cn (Q.O.); iamwhuang@njtech.edu.cn (W.H.); 2 Xi’an Institute of Flexible Electronics, Northwestern Polytechnical University, Xi’an 710072, China

**Keywords:** cellulose-based biomass, templated fabrication, metals and metal oxides, photocatalytic and bactericidal applications

## Abstract

In this paper, we report a simple, feasible and low-cost method to fabricate self-standing metallic materials using cellulose-based biomass as sacrificial templates. This process involves the impregnation of metallic precursors to the cellulose fibers of biomass templates and the transformation of the precursors to corresponding metals or metal oxides (as well as the removal of the cellulose framework) at an elevated temperature. The structures of the metallic materials as fabricated take the form of architectures of biomass templates (e.g., chromatography paper, medical absorbent cotton, catkins of reed, seed balls of oriental plane, and petals of peach blossom), and the various kinds of metals and metal oxides fabricated with these templates include silver, gold, anatase, cupric oxide, zinc oxide, etc. We have demonstrated photocatalytic and bactericidal applications of such metallic materials, and they should find more applications in electronics, catalysis, energy storage, biomedicine and so on.

## 1. Introduction

Recently, metallic materials with porous structures have gained great interest because of their excellent performance in catalysis, sensor, photonics, energy storage, and biomedicine [1,2,3,4,5]. The most commonly used method to prepare such materials is templated fabrication, a self-assembling process of introducing building blocks into a scaffold with designed configurations to achieve resulting products with replicated structures and controlled size distributions after sacrificing the original scaffold [6,7]. Different from most man-made templates, the plant-based cellulosic biomasses not only represent a sustainable biofuel resource [8], but are also the most abundant natural polymers on the Earth with hierarchical structures and diverse spatial morphologies [9]. In addition, biomass templates possess the following advantageous features: (1) low cost and versatility availing the abundance and variety of starting materials; (2) an inert property contributing to the reactions with multiple precursors and the synthesis of a wide range of materials from polymers to metallic materials [10,11]; (3) abundant inter- and intra-molecular hydrogen bonds allowing the direct deposition of metallic precursors [12]; and (4) adoptable ways of removing fibrous templates such as chemical etching [13] and thermal treatment [14] or of converting to carbon matrixes by carbonization in an oxygen-free atmosphere.

The pathway of a biomass-templated fabrication mainly involves three steps: the precursors coating on the surface of the fibrous network of the biomass templates, the conversion of the precursors to the resulting products, and the removal of cellulosic matrixes. Techniques such as the surface sol-gel process [15], atomic layer deposition [16], and chemical vapor deposition [17] have been verified effective methods for fabricating nanostructured materials by the replication of cellulosic substances. These strategies show excellent control in the structural organization of resultant analogues; however, they are adaptive for limited compounds and target products. On the other hand, the wet-chemistry method is simpler and more popular; it is suitable for diverse biomass templates and various metallic precursors [18,19]. Yet most metals fabricated through this wet-chemistry method are powders, and few endeavors have been reported for the preparation of self-standing metallic materials [20,21].

In this study, we fabricated self-standing metallic materials with hierarchical morphologies from biomass templates simply by heating in a furnace or burning in a flame. We explored the fabrication of metallic materials based on different cellulose-based biomass templates such as paper, cotton, catkins of reed, seed balls of oriental plane, and petals of peach blossom. The metallic materials reserve the replicated three-dimensional structures of the biomass templates. We have prepared a rich variety of metallic materials including silver, gold, anatase, cupric oxide, and zinc oxide through this biomass-templated method. We have further studied the photocatalytic and bactericidal applications of the metallic materials. We expect that this low-cost and simple method could be extended to produce more self-standing materials, which should find wide applications in catalysis, energy storage, biomedicine, etc.

## 2. Experimental Section

### 2.1. Materials

The Whatman^TM^ grade 1 chromatography paper (No. 3001-861) was purchased from Shanghai Jinpan Biolotech Co., Ltd. (Shanghai, China) The Kimberly-Clark^TM^ air-laid paper was purchased from Suzhou Weipusen Trading Co., Ltd (Suzhou, China). The medical absorbent cotton was purchased from Sangon Biotech (Shanghai) Co., Ltd (Shanghai, China). Catkins of reed, seed balls of oriental plane, and petals of peach blossom were gathered from natural plants. All chemicals used in the experiments were of analytical grade without further purification, and deionized water (18.2 MΩ cm at 25 °C) was obtained from Milli-Q system (Millipore, Bedford, MA, USA). Silver nitrate (AgNO_3_, AR, Reagent No.1 Factory of Shanghai Chemical Reagent Co., Ltd., Shanghai, China), titanium tetrachloride (TiCl_4_, AR, Shanghai Ling Feng Reagent Co., Ltd., Shanghai, China), cupric nitrate (Cu(NO_3_)_2_ 3H_2_O, AR, Adamas Reagent Co., Ltd., Shanghai, China), zinc nitrate hexahydrate (Zn(NO_3_)_2_ 6H_2_O, AR, Shanghai Titanchem Co., Ltd., Shanghai, China), and chloroauric acid (HAuCl_4_, AR, Reagent No.1 Factory of Shanghai Chemical Reagent Co., Ltd., Shanghai, China) were used as the metallic precursors. For the photocatalytical experiment, methyl orange (MO, AR, Tianjin Chemical Reagent Co., Ltd., Tianjin, China) was used as the organic pollution chemical, degraded with the existence of the paper-templated silver and sodium borohydride (NaBH_4_, AR, Sinopharm Chemical Reagent Co., Ltd., Shanghai, China). Luria-Bertani agar medium used in the antibacterial test was prepared by dissolving 5.0 g of tryphtone (Sangon Biotech (Shanghai) Co., Ltd., Shanghai, China), 2.5 g of yeast extract (Sangon Biotech (Shanghai) Co., Ltd., Shanghai, China), 5.0 g of sodium chloride (Xilong Scientific Co., Ltd., Shantou, China), and 7.5 g of agar (Shanghai Mackin Biochemical Co., Ltd., Shanghai, China) in 500 mL of deionized water.

### 2.2. Preparation of Metallic Materials

First, 50 μL of AgNO_3_ aqueous solution with different concentrations was dropped on the chromatography paper with a size of 2 cm × 2 cm. Then, the paper was placed on a glass slide and dried in a drying oven (DHG-9030A, Shanghai Yiheng Scientific Instruments Co., Ltd, Shanghai, China) at 35 °C for 5 min. Next, the paper was put in a muffle furnace (KSL-1200X-J, Hefei Kejing Materials Technology Co., Ltd., Hefei, China) and heated in the air at a temperature of 450 °C for 1 h. Finally, the samples were cooled to room temperature inside the furnace. Besides heating in a furnace, the silver was also fabricated by burning in a flame. First, 45 μL of 100 mM AgNO_3_ aqueous solution was dropped on the air-laid paper with a size of 2 cm × 5 cm. Then, the paper was dried in an oven followed by burning in the flame of an alcohol lamp within 3 s.

With the experimental conditions optimized using the paper template, other biomass templates such as medical absorbent cotton, seed balls of oriental plane, petals of peach blossom, and catkins of reed were also used to fabricate the silver. These templates were immerged in 4 M AgNO_3_ solution for 5 min with ultrasound and then removed from the solution with extra solution absorbed by paper pads. After being dried in an oven, they were heated in a furnace at a temperature of 450 °C for 1 h. In addition, the cotton was also employed as a template for fabricating other metallic materials using TiCl_4_, Cu(NO_3_)_2_, HAuCl_4_, and Zn(NO_3_)_2_ 6H_2_O as precursors.

The morphologies and compositions of the as-fabricated metallic materials were characterized using a scanning electron microscope (SEM, JSM-7800F, JEOL Ltd., Tokyo, Japan), energy dispersive spectroscopy (EDS, X-Max, Oxford Instruments, Oxford, UK), and X-ray diffraction (XRD, Smartlab 3KW, Rigaku Corp.,Tokyo, Japan). All digital photographs were captured with digital camera (EOS 700D, Canon (China) Co., Ltd., Beijing, China).

### 2.3. Optical Catalysis Evaluations

The photocatalytic property of the paper-templated silver was evaluated by the degradation of the methyl orange (MO) solution [22]. A piece of the silver with a weight of 0.0089 g was used as the catalyst; it was wrapped in a stainless steel mesh to form a “tea bag”. Then, the “tea bag” was put into a beaker with a mixed solution of 40 mL 8 × 10^−5^ M MO and 5 mL 0.2 M NaBH_4_. The beaker was sealed with parafilm and the mixed solution was irradiated by a 300 W Xe arc lamp (PLS-SXE300, Beijing Perfectlight Technology Co., Ltd., Beijing, China) at room temperature with a magnetic stirrer for 1 h. The light source was put on one side of the beaker with a fixed distance of 8 cm between the beaker and the light source. During the irradiating process, 1.5 mL of the solution was pipetted out from the beaker every 15 min and kept in the dark. The absorption spectra of the pipetted solutions were obtained with a Ultraviolet-visible (UV-vis) spectrophotometer (Shimadzu UV-1750, Shimadzu Corp., Kyoto, Japan).

### 2.4. Antibacterial Test

*Escherichia coli* (*E. coli*, ATCC 25922) and Methicillin-resistant Staphylococcus aureus (MRSA, ATCC BAA40) were obtained from the American Type Culture Collection (ATCC, Manassas, VA, USA). The antimicrobial activity of the paper-templated silver was evaluated against the Gram-positive bacteria MRSA and the Gram-negative bacteria *E. coli*, using the agar disk diffusion method [23]. The Luria-Bertani (LB) agar medium (pH 7.4) was prepared and autoclaved. Then, the agar medium was cast into a petri dish and cooled down to room temperature. Next, a single bacteria colony was selected into liquid LB medium and cultivated at 37 °C to the logarithmic phase. The optical density of the bacteria suspension was adjusted to 0.05, and a 50 μL bacteria suspension was transferred to a fresh agar plate uniformly. Four testing samples with a diameter of 0.7 cm including the paper, the paper-templated silver (the amount of silver was 0.0053 g), the purchased silver foil, and the reused paper-templated silver were placed in the petri dish. The whole dish was incubated in an incubator (GRP-9160, Shanghai Senxin Experiment Instrument Co., Ltd., Shanghai, China) at 37 °C for 12 h, after which the dish was removed from the incubator and the diameters of the inhibition zone with respect to the samples were gauged with a ruler.

## 3. Results and Discussion

### 3.1. Paper-Templated Fabrication

**Figure 1:**
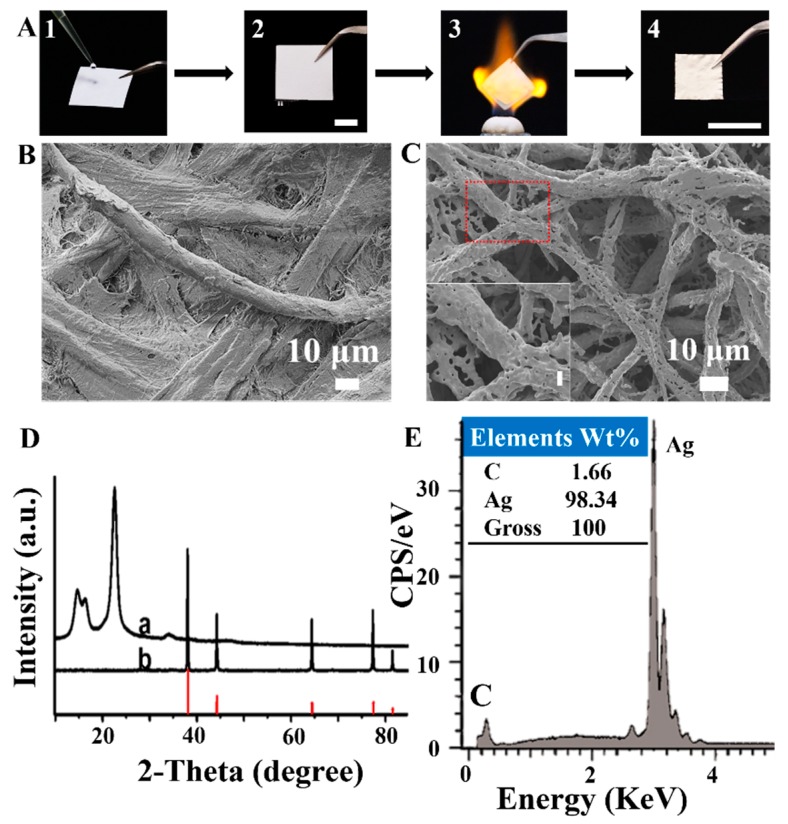
Fabrication of the paper-templated silver. (**A**) Digital photographs showing the fabrication process of silver from paper template (scale bar: 1 cm). SEM images of (**B**) the paper template and (**C**) the silver fabricated from the paper template. Inset: enlarged SEM image of the red square with a scale bar of 10 μm. (**D**) XRD patterns of (a) the paper and (b) the paper-templated silver. The red lines inserted are a PDF card of silver (PDF#04-0783). (**E**) Energy dispersive spectroscopy (EDS) spectrum of the paper-templated silver. CPS: counts per second.

We initially chose paper as a model template to optimize the experimental conditions of this biomass-templated method for fabricating free-standing metallic materials because paper is cheap and easily available. The procedure of this paper-templated fabrication is shown in Figure 1A: (1) the precursor solution was dropped onto a slice of paper (here, we used an aqueous solution of AgNO_3_ as an example); (2) the paper was dried in a drying oven; (3) the paper was heated in a furnace or burned in a flame; and (4) a self-standing foil of silver was obtained.

We have investigated the factors that influenced the paper-templated fabrication, including the type of paper used as templates, the volume and concentration of the silver nitrate solution added on the paper, and the temperature of the heat treatment, etc. For example, we compared different types of paper (e.g., printing paper, newspaper, chromatography paper, and air-laid paper) as templates and chose Whatman grade 1 chromatography paper because it is hydrophilic, pure, homogeneous, and more uniform in structure than other papers [24]. We fixed the volume of the precursor solution in order to load each paper with an equal amount of precursors: the optimal volume of the solution was 50 μL for the paper with a size of 2 cm × 2 cm. Also, the optimized concentration of the silver nitrate solution was 4.0 M, with a heating temperature of 450 °C for 1 h to fabricate self-standing silver.

With the above optimized experimental conditions, the as-fabricated paper-templated silver retained the shape of the paper with the size shrunken from 2 cm × 2 cm to 1 cm × 1 cm (Figure 1A). The morphology of the paper-templated silver (Figure 1C) resembled the original one of the paper template with architectures composed of fibers (Figure 1B). The silver precursors, silver nitrate, added to the paper were decomposed to silver (Figure 1D) [25], comprised primarily (>98%) of Ag (Figure 1E). Moreover, the paper-templated silver was permeable to gases and liquids and revealed an excellent conductivity with a low resistance (Figure S1A,C), showing its potential for catalysis and energy applications.

Besides heating in a furnace, we have also fabricated metallic materials by burning in a flame. For example, air-laid paper was made wet by dropping 100 mM AgNO_3_ solution and drying in an oven before burning. Paper-templated silver with wires having diameters of ~500 nm was produced (Figure S2B). The flame burning method could not only burn off paper templates but also reduce silver nitrate to silver at the same time (Figure S2C). Although the silver fabricated by the flame burning method was relatively impure, wrinkling, thin, and brittle, this method has shown great advantages in its briefness and rapidness (within 3 s) and had the potential for a large-scale preparation of silver materials with further improvement.

### 3.2. Biomass-Templated Fabrication

**Figure 2:**
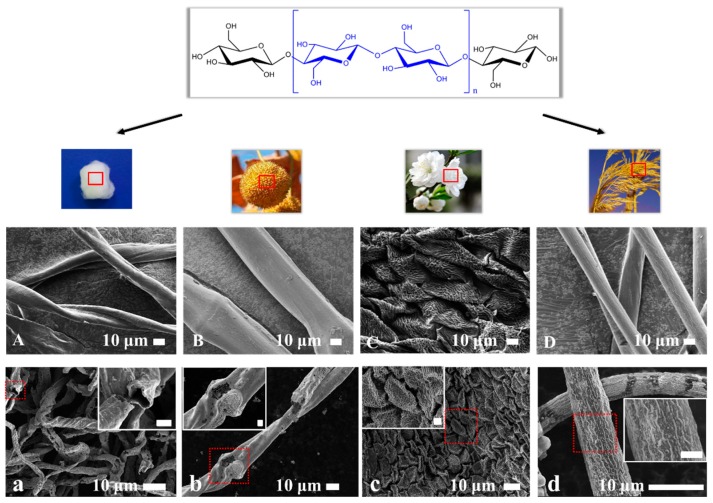
Silver fabricated from different cellulose-based biomass templates: (**A**,**a**) medical absorbent cotton, (**B**,**b**) seed balls of oriental plane, (**C**,**c**) petals of peach blossom, and (**D**,**d**) catkins of reed. The above shows the molecular structure of the cellulose and digital photographs of the cellulose-based templates. SEM images of (**A**–**D**) original biomass templates and (**a**–**d**) silver fabricated from the templates. The insets in the last row are enlarged SEM images of the red square, and all the scale bars are 2 μm.

Besides the paper template, this templated method can be extended to other cellulose-based biomass templates. For example, we have used medical absorbent cotton, seed balls of oriental plane, petals of peach blossom, and catkins of reed as templates to fabricate self-standing silver with hierarchical morphologies and different spatial distributions (Figure 2, Figures S3 and S4). The size of the as-fabricated silver was smaller than that of their respective biomass templates, due to the size shrinkage in paper-templated fabrication. The microstructures of the silver (Figure 2a–d) preserved the key features of their original biomass templates (Figure 2A–D): hollow silver spiral tubes fabricated from medical absorbent cotton, silver wires fabricated from seed balls of oriental plane and catkins of reed, and fish scale-like silver fabricated from petals of peach blossom. Different from the other three biomass templates, the petals of peach blossom contained reductive compositions which would lead to the reduction of silver ions on the surface of cellulose and in solution [26]. In all, more, if not any, cellulose-based biomass templates are expected to be used to fabricate self-standing metallic materials by this simple templated method.

### 3.3. Fabrication of Other Metallic Materials

**Figure 3:**
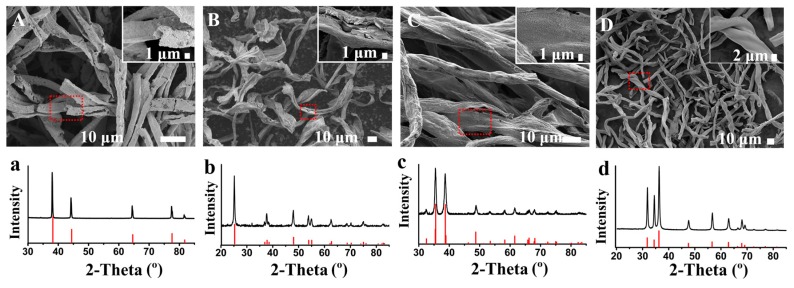
Various metallic materials fabricated from the template of medical absorbent cotton: (**A**,**a**) gold, (**B**,**b**) anatase, (**C**,**c**) cupric oxide, and (**D**,**d**) zinc oxide. (**A**–**D**) SEM images and (**a**–**d**) XRD patterns of the metallic materials. The red lines are PDF cards of (**a**) gold (PDF#04-0784), (**b**) anatase (PDF#21-1272), (**c**) tenorite (PDF#48-1548), and (**d**) zincite (PDF#36-1451).

In addition to silver, this templated method can also be extended to fabricate many other metals and metal oxides. For example, we have used medical absorbent cotton as a template to fabricate gold, anatase, cupric oxide, and zinc oxide. Similar to the as-fabricated silver, these metals and metal oxides also retained the fiber structure of the cotton template (Figure 3A–D). The XRD patterns of these metals and metal oxides contain no impurity peaks, with all characteristic peaks being in agreement with the standard XRD pattern, implying the high purity of the gold, anatase, cupric oxide, and zinc oxide (Figure 3a–d). In these as-fabricated metallic materials, metal oxides tend to be more fragile than metals. In all, more, if not any, metals and metal oxides are expected to be fabricated by this biomass-templated method, reserving the replicated hierarchical structures of the biomass templates.

### 3.4. Photocatalytic and Bactericidal Applications

We further studied the photocatalytic and bactericidal applications of the metallic materials, using the paper-templated silver as an example. Currently, the silver used in optical catalysis with prominent efficiency is commonly in the form of nanoparticles. Although photocatalysis in nanoscale with a large surface to volume ratio exhibits tremendous advantages in remediate pollutants, nanoparticles show limitations in the stability, recovery, regeneration, and removal from solution after a catalytic reaction. Compared to silver nanoparticles, the paper-templated silver, also with a large surface to volume ratio [20], has shown an advantage in detaching from the solution after degradation. The photocatalytic properties of the paper-templated silver were demonstrated by the degradation of the MO solution, where MO is an aromatic azo dye that is toxic and harmful to both humans and the environment. Because of the destruction of the conjugated chromophore in optical catalysis, the dye can be reduced to a colorless hydrazine derivative with a change of the maximum absorption from λ_max_ = 463 nm to λ_max_ = 247 nm. The paper-templated silver is not fragile, so we could pack it into a tea bag-like stainless steel mesh (Figure S5A), making it easier to be separated from the solution after the degradation reaction.

**Figure 4:**
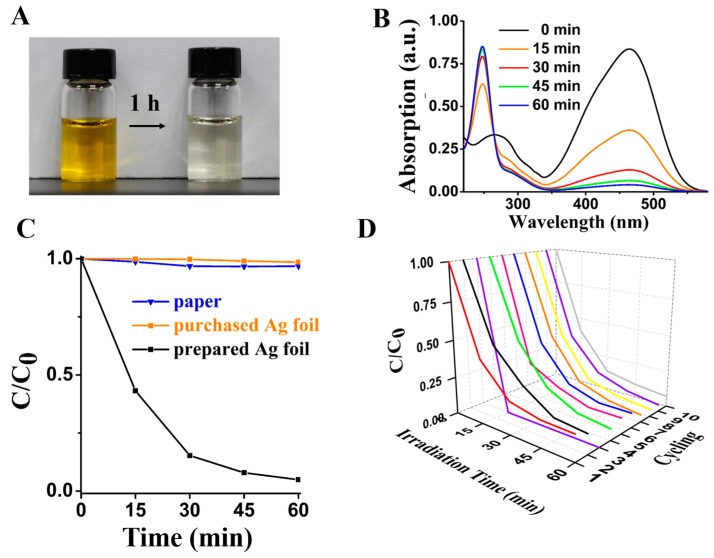
Photocatalytic performance of the paper-templated silver. (**A**) A digital photograph of the MO solution before and after a one-hour irradiation in the presence of the paper-templated silver. (**B**) The absorption-wavelength graph of the MO solutions every 15 min. (**C**) Plot of C/C_0_ vs. time in the presence of paper (blue), pure Ag (orange) and the paper-templated silver (dark), where C is the concentration of the MO solution at different times and C_0_ is the concentration of the original MO solution. (**D**) Plot of C/C_0_ in the presence of the paper-templated silver vs. time for 10 cycles.

We can clearly see the discoloration of the MO solution after the irradiation for 1 h (Figure 4A). We investigated the photocatalytic process by obtaining MO solutions every 15 min (Figure S5B). The UV-vis absorbance shows that the paper-templated silver has a significant degradation efficiency of over 90% for the MO solution in 1 h (Figure 4B). Compared to other reports, the paper-templated silver showed an excellent photocatalytic activity with a shorter irradiation time (Table S1). Besides, the degradation rate decreases with time because of the reduction of most MO in solution and because there is less opportunity for contact between the catalyst and MO molecules [27]. The degradation properties of paper and pure silver were compared with those of the paper-templated silver (Figure 4C), showing no catalytic property. The purchased Ag foil is bulky material with relatively smooth surfaces (Figure S6A), whereas the paper-templated silver retains porous structure of the paper (Figure S6B), showing much larger surface to volume ratio than that of the commercial Ag foil. The reusability of the paper-templated silver was evaluated by using the same silver in ten repeated photocatalytic degradation experiments of the MO solution. After ten experiments, the silver showed no weight loss and the degradation efficiency of each test was higher than 90% (Figure 4D). When the paper-templated silver was stored in the MO solution for 1 h under dark condition, the color of the solution did not change (Figure S7).

**Figure 5:**
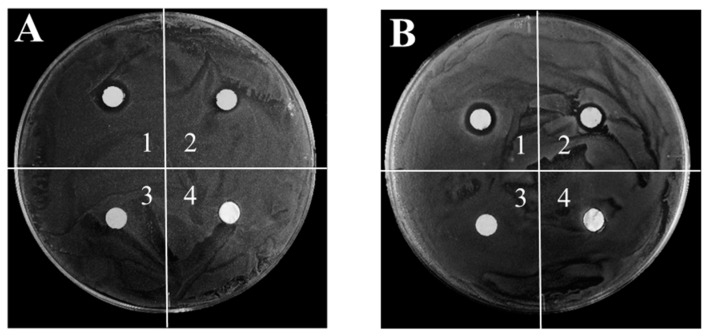
Antibacterial property of the paper-templated silver. Digital photographs of four samples against (**A**) MRSA and (**B**) *E. coli* in agar dishes. The four samples are (1) the paper-templated silver, (2) the paper-templated silver used for the second time, (3) paper, and (4) pure silver foil.

We also evaluated the antibacterial ability of the paper-templated silver against the Gram-positive bacteria MRSA and Gram-negative bacteria *E. coli* by the agar disk diffusion method. The antibacterial effect of the paper-templated silver compared with two control samples (the paper and the purchased pure silver foil) was determined by measuring the size of each inhibition zone, which presented as the diameter of the area of no bacterial growth minus the diameter of the sample [28]. As is clearly shown in Figure 5 and Figure S8, the MRSA colony and *E. coli* colony seeded in the dishes grew on the paper and the purchased silver foils after incubation at 37 °C for 12 h. The paper and the purchased silver foil showed no antibacterial activity. On the other hand, clearly defined bacterial free zones around the paper-templated silver and reused paper-templated silver against MRSA and *E. coli* are revealed in Figure 5 and Figure S8. The inhibition zone of the newly prepared paper-templated silver was larger than the zone of reused silver, indicating a higher antibacterial effect of the newly prepared silver. According to the Standard Antibacterial test ‘SNV 195920-1992’ [23], an antibacterial material is considered as “good” when the size of the inhibition zone is larger than 1 mm. The quantitative results of the size of the growth inhibition zones are shown in Table 1. The sizes of the growth inhibition zones around the newly prepared paper-templated silver and the paper-templated silver used for the second time against MRSA and *E. coli* were all larger than 1 mm, showing that the bactericidal effect of the paper-templated silver used twice is “good”. The inhibition zone was very small for the second time because the release rate of silver ions from the paper-templated silver gradually decreased with time [29], and because the surface of the paper-templated silver might be partially oxidized after the first use. The excellent antibacterial ability of the paper-templated silver results from the existence of silver and the release of silver ions, though the antibacterial efficiency of the paper-templated silver used for the second time was lower than that of the silver used the first time. Compared to previous reports, the paper-templated silver showed lower antimicrobial activity than that of the silver nanoparticles [30,31], probably due to a smaller surface to volume ratio than that of the silver nanoparticles [32].

## 4. Conclusions

To summarize, this article showcases an easy method for preparing various metallic materials based on cellulosic biomass templates. The superiority of the biomass templates over other templates is in its physicochemical properties, extensive resource, and hierarchical morphologies. A high-temperature treatment after the impregnation of metallic precursors to cellulose fibers of biomass templates contributes to both the production of metallic materials from precursors and the simultaneous removal of original biomass templates. The combination of the widely available biomass templates and the simple high-temperature treatment sheds new light on the syntheses of functional macro-/nano-structured metallic materials with diverse morphologies. Moreover, this low-cost method shows promise for being implemented in the large-scale synthesis of metallic materials ranging from metals (e.g., silver and gold) to metal oxides (e.g., anatase, cupric oxide and zinc oxide). In addition, we have also proved that the flame burning is a very simple alternative approach to the fabrication of biomass-templated metallic materials. 

We have mainly explored the properties and applications of the paper-templated metals, as examples of the biomass-templated metallic materials. First, the paper-templated silver and gold were in the form of sheets and conductive with a low resistance, suggesting their potential applications as electrodes in electronics and optoelectronics. Second, the paper-templated silver has shown excellent photocatalytic effects (>90%) in more than ten cycles of repeated optical degradation tests and can be easily separated from the degradation solution. Third, the paper-templated silver presents outstanding antibacterial abilities against the Gram-positive bacteria MRSA and Gram-negative bacteria *E. coli*, even when reused for the second time. With all the properties exemplified here, the metallic materials, especially the paper-templated noble metals fabricated through this biomass-templated method, could be employed as promising candidates in antibacterial, catalytic, sensing and electronic applications.

## Figures and Tables

**Table 1:** The size of the inhibition zones of four samples against the Gram-positive bacteria MRSA and Gram-negative bacteria *E. coli* (n = 5).

Sample	Paper-Templated Silver (mm)	Paper-Templated Silver Used for Two Times (mm)		Paper (mm)	Pure Silver Foil (mm)
		The First Time	The Second Time		
MRSA	3.6 ± 0.6	3.6 ± 0.7	1.4 ± 0.3	0	0
*E. coli*	3.6 ± 0.9	3.6 ± 0.8	1.3 ± 0.6	0	0

## Supplementary Materials

The following are available online at https://www.mdpi.com/1996-1944/12/8/1271/s1. Figure S1: The low resistance and excellent conductivity of paper-templated (A,C) silver and (B,D) gold. Figure S2: The air-laid paper-templated silver fabricated by flame burning. (A) SEM image showing three-dimensional network of the paper–templated silver. An enlarged SEM image of the red square is shown in (B). (C) XRD pattern of the paper–templated silver (black) and the PDF card of silver (PDF#04-0783) (red). Figure S3: Digital photographs of silver fabricated from biomass templates: (A) medical absorbent cotton, (B) seed balls of oriental plane and (C) catkins of reed. Figure S4: XRD patterns of silver fabricated from biomass templates: medical absorbent cotton (black), seed balls of oriental plane (blue), petals of peachblossom (pink), and catkins of reed (purple) with PDF card of silver (PDF#04-0783) (red). Figure S5: Photocatalytic performance of paper-templated silver. (A) The tea bag-like stainless steel mesh for containing the paper-templated silver in the photocatalytic test. (B) Digital photograph of MO solutions after irradiating for every 15 minutes in the presence of the paper-templated silver. (C) Plot of C/C_0_ vs time with and without NaBH_4_ in the presence of the paper-templated silver. Figure S6: SEM images of (A) the purchased Ag foil and (B) paper-templated silver. Figure S7: Catalytic performance of paper-templated silver under dark condition. (A) A digital photograph of MO solutions in every 15 minutes without paper-templated silver. (B) A digital photograph of MO solutions in every 15 minutes in the presence of the paper-templated silver. Figure S8: More digital photographs of four samples against (A) MRSA and (B) E. coli in agar dishes. The four samples are (1) the paper-templated silver, (2) the paper-templated silver used for the second time, (3) pure silver foil, and (4) paper. Table S1: The degradation efficiency of paper-templated silver compared with other reports.
