# Supplementary material for: Biomass-Templated Fabrication of Metallic Materials for Photocatalytic and Bactericidal Applications
Source: Materials (Basel). 2019 Apr 18;12(8):1271. doi: 10.3390/ma12081271 (PMC6514999; doi:10.3390/ma12081271)
Supplement: Supplementary file 1 [file materials-12-01271-s001.pdf]

materials

Supplementary Materials

Biomass-Templated Fabrication of Metallic Materials for Photocatalytic and Bactericidal Applications

Xueying Guo [1], Qianqian Wang [1], Qiongyu Lai [1], Qiran Ouyang [1], Peng Li [1,2,]* Hai-Dong Yu [1,2,]*and Wei Huang [1,2]

[1] Institute of Advanced Materials (IAM) & Key Laboratory of Flexible Electronics (KLOFE), Jiangsu National Synergetic Innovation Center for Advanced Materials (SICAM), Nanjing Tech University (NanjingTech), Nanjing 211816, China; iamxyguo@njtech.edu.cn (X.G.); qqwang0717@njtech.edu.cn (Q.W.); laiqionyu03@njtech.edu.cn (Q.L.); iamoyqr@njtech.edu.cn (Q.O.); iamwhuang@njtech.edu.cn (W.H.)

[2] Xi'an Institute of Flexible Electronics, Northwestern Polytechnical University, Xi'an 710072, China

* Correspondence: iamhdyu@njtech.edu.cn (H.-D.Y.); iampli@njtech.edu.cn (P.L.)

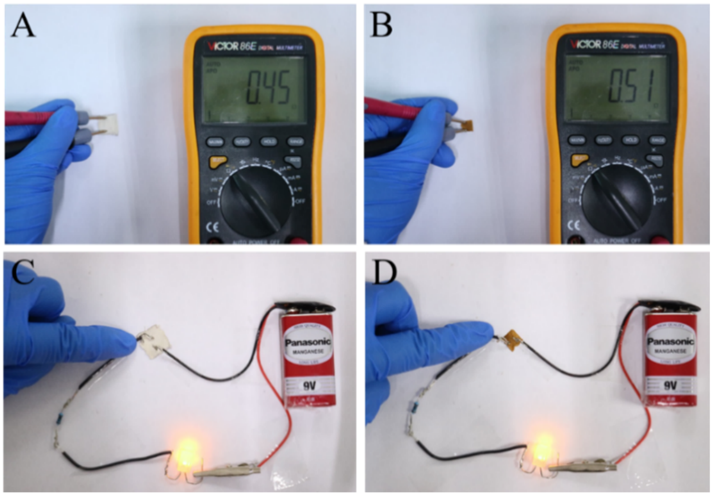

Figure S1. The low resistance and excellent conductivity of paper-templated (A,C) silver and (B,D) gold.

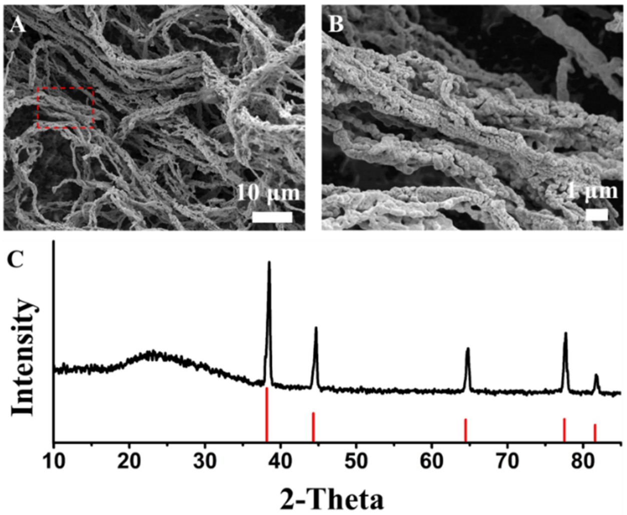

Figure S2. The air-laid paper-templated silver fabricated by flame burning. (A) SEM image showing three-dimensional network of the paper–templated silver. An enlarged SEM image of the red square is shown in (B). (C) XRD pattern of the paper–templated silver (black) and the PDF card of silver (PDF#04-0783) (red).

The broad peak of about 23 degrees in XRD (Figure S2C) is the peak of carbon. The residual carbon in the paper–templated silver prepared by burning in a flame is more than that in the paper–templated silver prepared by heating in a furnace.

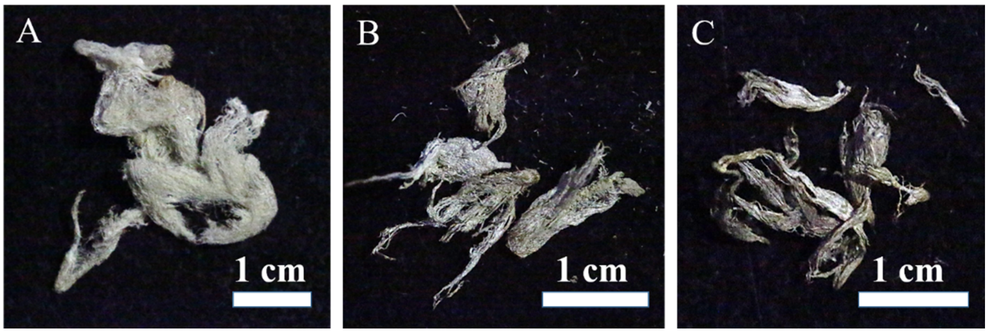

Figure S3. Digital photographs of silver fabricated from biomass templates: (A) medical absorbent cotton, (B) seed balls of oriental plane and (C) catkins of reed.

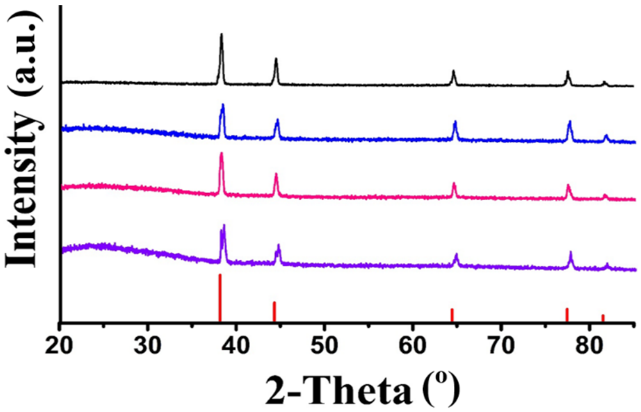

Figure S4. XRD patterns of silver fabricated from biomass templates: medical absorbent cotton (black), seed balls of oriental plane (blue), petals of peachblossom (pink), and catkins of reed (purple) with PDF card of silver (PDF#04-0783) (red).

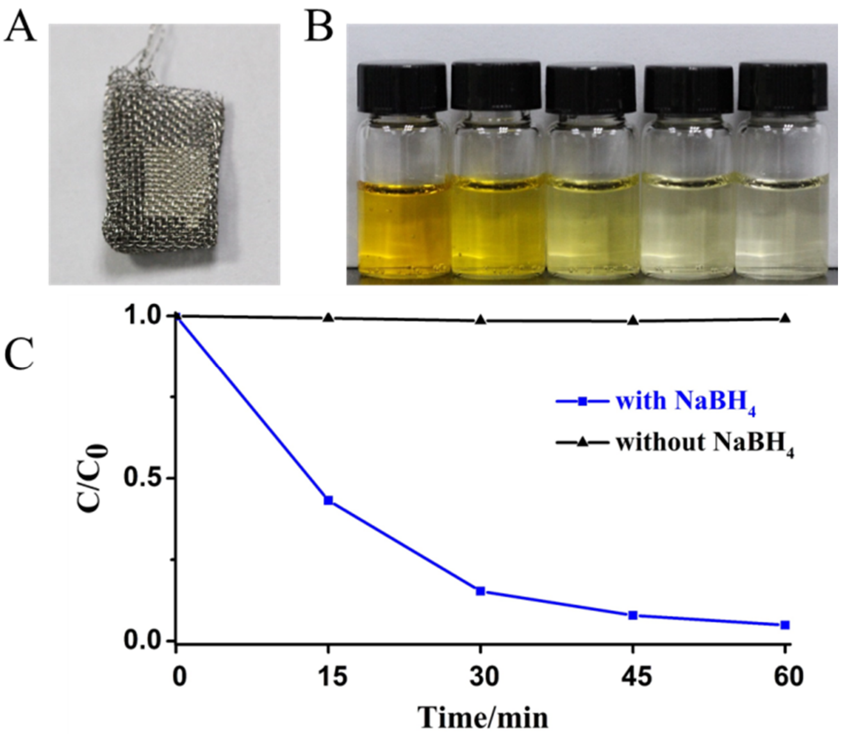

Figure S5. Photocatalytic performance of paper-templated silver. (A) The tea bag-like stainless steel mesh for containing the paper-templated silver in the photocatalytic test. (B) Digital photograph of MO solutions after irradiating for every 15 minutes in the presence of the paper-templated silver. (C) Plot of C/C_0 vs time with and without $NaBH_4$ in the presence of the paper-templated silver.

In the photocatalytic reaction, $NaBH_4$ was used to reduce MO (Figure S5C), while the addition of silver could accelerate the reaction.

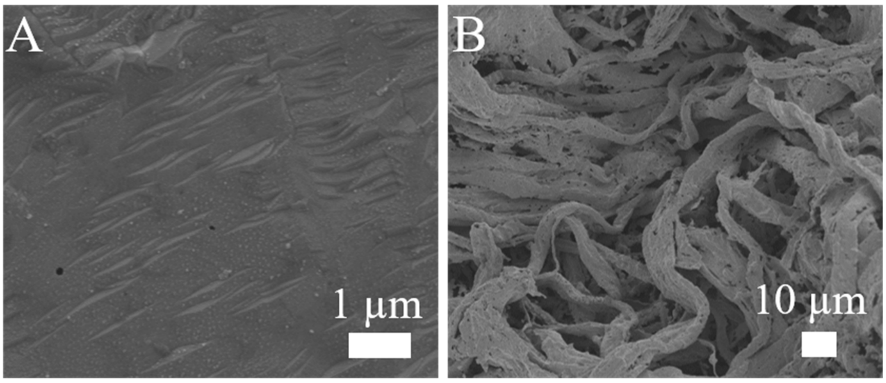

Figure S6. SEM images of (A) the purchased Ag foil and (B) paper-templated silver.

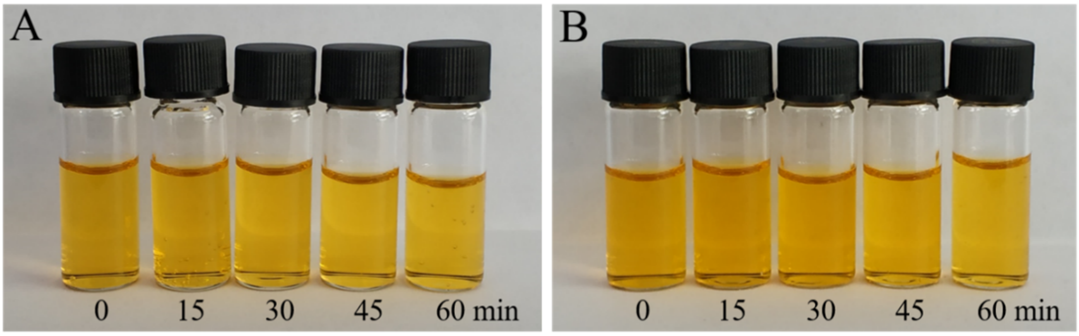

Figure S7. Catalytic performance of paper-templated silver under dark condition. (A) A digital photograph of MO solutions in every 15 minutes without paper-templated silver. (B) A digital photograph of MO solutions in every 15 minutes in the presence of the paper-templated silver.

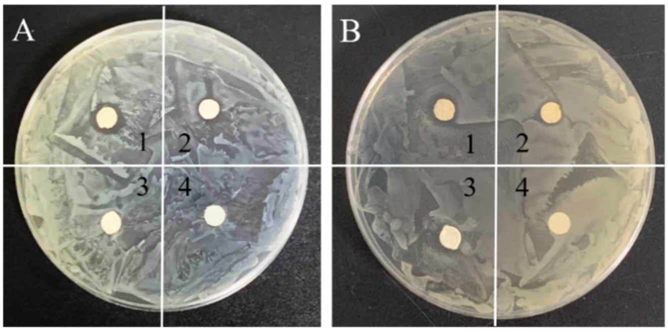

Figure S8. More digital photographs of four samples against (A) MRSA and (B) E. coli in agar dishes. The four samples are (1) the paper-templated silver, (2) the paper-templated silver used for the second time, (3) pure silver foil, and (4) paper.

Table S1. The degradation efficiency of paper-templated silver compared with other reports.

Photocatalyst	MO Concentration (mg/L)	Degradation Ratio (%)	Irradiation Time (min)	Light Source	References
AgNPs	2.5	90	240	Xe lamp (200 W)	[1]
Core-shell $Cu@Cu_2O$	20	90	100	Sun light	[2]
$Cu_2O@Cu$	10	90	120	Tungsten lamp (40 W)	[3]
Cu_2O nanocube	20	83.6	120	Xe lamp (300 W)	[4]
Paper-templated silver	23	90	60	Xe lamp (300 W)	This work

References

1. Park, H.; Hira, S. A.; Muthuchamy, N.; Park, S.; Park, K. H., Synthesis of silver nanostructures in ionic liquid media and their application to photodegradation of methyl orange. Nanomater. Nanotechnol. 2019, 9.
2. Kou, T.; Jin, C.; Zhang, C.; Sun, J.; Zhang, Z., Nanoporous core-shell $Cu@Cu_2O$ nanocomposites with superior photocatalytic properties towards the degradation of methyl orange. RSC Adv. 2012, 2, 12636-12643.
3. Zhou, B.; Liu, Z.; Wang, H.; Yang, Y.; Su, W., Experimental study on photocatalytic activity of Cu_2O/Cu nanocomposites under visible light. Catal. Lett. 2009, 132, 75-80.
4. Sun, W.; Sun, W.; Zhuo, Y.; Chu, Y., Facile synthesis of Cu_2O nanocube/polycarbazole composites and their high visible-light photocatalytic properties. J. Solid State Chem. 2011, 184, 1638-1643.

© 2019 by the authors. Licensee MDPI, Basel, Switzerland. This article is an open access article distributed under the terms and conditions of the Creative Commons Attribution (CC BY) license (http://creativecommons.org/licenses/by/4.0/).
